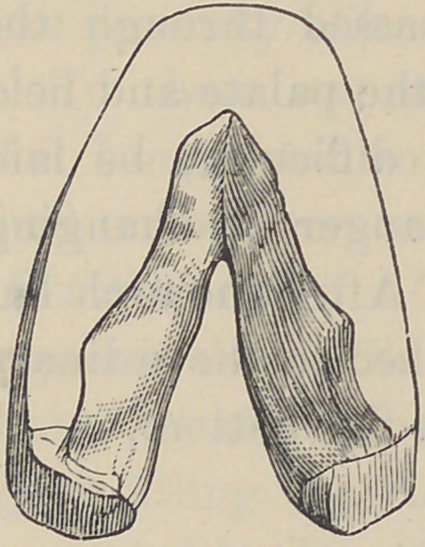# Artificial Vela for Congenital Cleft of the Palate

**Published:** 1888-09

**Authors:** C. S. Case

**Affiliations:** Jackson, Mich.


### Artificial Vela for Congenital Cleft of the Palate.

BY C. S. CASE, M.D., D.D.S., JACKSON, MICH.

[Read before the Michigan State Dental Association, held at Ann Arbor, Michigan, March 20, 1888.]

I am reported to have said at the December meeting of the Chicago Dental Society, that "I did not think the making and introduction of artificial vela difficult." I am aware that I made some strong statements relative to the possibilities of making this department of our art as simple mechanically as other portions of dentistry, for it seemed to me that the paper upon the subject by Dr. E. D. Swain, and much of the discussion which followed was calculated to magnify its difficulties and hold it beyond the reach of the ordinary practitioner; whereas I can but believe that any good dentist who will seek the information and means at his disposal may hope to produce a very satisfactory cleft palate instrument, and one that will improve if not entirely restore the function of articulate speech.

I have two patients with wide clefts through the entire soft and hard palate—both wearing soft rubber vela—who speak with perfect distinctness of articulation; and one of them with so much freedom from the usual nasal tone that a stranger hearing him talk would not imagine there was any physical abnormality. An incident occurred recently which will serve to illustrate this. About three months ago I inserted a cleft palate instrument for Miss H., a resident of Chicago, and being a young lady of considerable intelligence I employed her temporarily as bookkeeper and amanuensis, in order to give her all the advantages I could in vocal articulation.

The cleft, which you will observe by the model of her mouth which I hold in my hand, is a small one extending only partially through the soft palate. At first I pronounced it a case for plastic surgery, and upon being told that such an operation had been performed twice, I found upon further examination that cicatritial contraction from these operations would render it impossible for the velum palati to close the vaso-pharyngeal passage even though perfect union of the bifurcated portions were permanently established.

You all understand that every word we utter which does not contain the sounds of m and n, is made with the pharyngeal passage to the nose entirely closed by the velum palati acting in conjunction with the pharyngeal muscles; and if this object can not be fully accomplished either by surgery or artificial means, vocal articulation will not be materially improved.

Notwithstanding the smallness of the cleft in this case, her speech was as imperfect as the most pronounced case of congenital cleft palate I ever saw. This I afterwards found to be largely due to the failure of cultivating the organs at her command. Being an exceedingly sensitive person her parents and friends had refrained from correcting or assisting her in any way. By the aid of the artificial velum and subsequent vocal instruction, there has been a gradual marked improvement though not nearly so rapid or complete as in the case of the young man whom I had before you three years ago, who, at that time, had worn an artificial palate but two months.

About four weeks ago Walter S., one of the patients who speaks so perfectly—being on a visit to his parents, who reside in Jackson—called upon me unexpectedly. I was surprised at the improvement he had made in the last year in his enunciation and tone, and not being able to attend to him at once I asked him to sit in the reception room and talk freely with a companion who came with him, and to speak as distinctly as he could, at the same time explaining my reasons. This he did for about half an hour, while Miss H. sat at work a short distance from them. After they had gone I asked her if she noticed anything peculiar about either of the young men. "Only that one of them talked a great deal and told many amusing things which she could not help but listen to." Imagine her surprise when I told her that this was the model of his mouth. You will see by examining it, that it represents a wide cleft extending completely through the soft and hard palate.

Such success, however, is not always attainable even with a most perfect cleft palate appliance, and while it will largely depend upon the scientific perfection of the vellum or obdurator and the subsequent training in its use, more will be due to the capabilities and perseverance of the patient. The operator, moreover, should be able to appreciate the various powers of the rudimentary muscles in their efforts to make use of the instrument, and, if necessary, perfect its size and shape according to their demands in order that it be made to do its best work.

I do not wish to be understood as claiming that all these things can be accomplished by the novice without difficulty or even without special ability, skill, and experience. To make a wagon or a set of teeth requires intelligence and skill, yet these things become simple to one who will put the requisite amount of work into a careful study of the principles involved and acquire the successive steps in their mechanical construction, and what I claim, is, that artificial vela and obdurators for congenital cleft of the palate are by no means beyond the possibilities of these simple demands.

The difficulty which the dental profession labors under to-day, is the need in text-books of proper and sufficient instruction relative to the practical steps of this work. To those who have not made the attempt to follow the directions of some text-book in the construction of their first case, such a statement may seem improbable, and especially to a casual enquirer who turns over the pages of "Kingsley's Oral Deformities" and sees the space given to this department with the array of illustrations and descriptions. Yet were that one to attempt to use it as a guide in the construction of means for producing that which is so perfectly described, *i. e.*, the Kingsley velum, he would find it sadly deficient in specific directions for practical procedure; and now the same thing can be said of the *American System of Dentistry.*

It would seem as if text-books which pretend to teach this department of dentistry should fully describe the successive steps in the operation, especially in regard to that which taxes so greatly the ingenuity of the beginner, and for the want of proper working directions too often results in a bungling and useless affair that leads him to resolve never to try it again.

Some years ago I read a paper before this body, which was subsequently published in the *Cosmos*, describing "A Method for Producing the Kingsley Velum."* By this method the work can be accomplished with such ease, rapidity, and mechanical nicety, and when finished is so completely under the control of the operator, to change the shape, size, and thickness of the artificial palate that I can but repeat, "I do not think the making and introduction of artificial vela more difficult than many other portions of dentistry."

There is one thing, especially which I wish to deprecate, and that is the tendency of writers to overestimate the difficulties of taking an impression for an ordinary artificial velum. The space that is usually devoted to details of various methods and the introduction and exhibition of paraphernalia, implies the necessity of duplicating in the model, much that is of no practical use whatever, and involves difficulties, that to some, seem almost insurmountable. In the *American System of Dentistry* is elaborately described a very ingenious method of forcing plaster into the nasal portion of the cleft through a rubber tube by the use of a piston syringe. A similar machine was shown and its use described by Dr. Swain, and I suppose every one, I, among the rest, has some pet scheme for taking impressions of these difficult and, unless it be in rare and anomalous cases, unnecessary parts.

With the view of determining the requisites of an impression, let us examine the needful shape of a cleft palate instrument, the object of which is to securely close the palatal cleft and possess a movable attachment, by the aid of which the patient can at will prevent sound from passing through the nares.

If you wish to make a soft rubber velum with a plate upon which it can be buttoned so as to retain it in position, you will first see that the mouth is of such shape as to firmly sustain a supporting plate, (and it will be a very odd mouth indeed which will not, either by clasps or other means, retain some kind of a plate.) This favorably determined, an impression of the nasal border of the cleft with a view of producing over-lapping and sustaining extensions to the rubber palate, will be entirely unnec-

* The same has been revised and recently published in the *Archives*.—ED.

essary; in fact, the oral or inferior border of the cleft, and that portion of the roof of the mouth over which the lateral palate wings are to extend, are the only parts that are absolutely essential to duplicate in the first model.

Dr. Kingsley says: "It is only when the floor of the nares is used to support the palate that it becomes necessary to obtain a more complicated impression, one that will represent not only a portion of the buccal cavity, but all the super-jacent nasal cavity."

The only object that I have ever seen for extending the instrument any distance into the cleft of the hard palate, is to obtain a vertical thickness of the rubber palate and consequent rigidity, sufficient to support the velum when buttoned to the sustaining plate. This can be better shown by a drawing.

Fig. 1. represents a section through the mesial line of a soft rubber palate A, buttoned to the sustaining plate B, by the pin C. It will be noticed that the anterior end of the palate, being thick, will have a longer and more rigid bearing upon the pin of the plate with increased powers for sustaining the velum, to which it should gradually taper in thickness.

The palatal portion of the instrument should fit in the roof of the mouth as perfectly as an artificial denture, and also immediately upon entering between the borders of the cleft in the hard palate. A model that will fulfill these requisites is all that is absolutely necessary, and certainly not very difficult to obtain. See Fig. 2.

The remaining portion of the instrument—the velum—being subjected to the constantly varying positions of the bifurcated velum palati and pharyngeal muscles, is not intended to *fit* these parts even were it possible, but rather is shaped so as to be *adapted* to their use. This can be accomplished only by repeatedly trying the model of the velum in the mouth; and even after completion the possibility of readily changing its shape according to demands that are not at first recognized, is a matter of no little importance.

When the cleft is confined to the soft palate or extends but a short distance into the hard palate an impression of the roof of the mouth and the border of the anterior portion of the fissure will be sufficient, and not more difficult to obtain than an ordinary impression. The palatal portion of the artificial palate may be made to cover the same extent of surface in the roof of the mouth as in other cases, but instead of depending upon the supporting plate to sustain the velum in position, atmospheric pressure can be made to do this work most perfectly; the only advantage in this case of the supporting plate being to prevent the plate from slipping into the throat in an unguarded moment, and also to facilitate its insertion and removal.

In taking the final impression for the supporting plate, I prefer to wait until the velum is completed and ready for the mouth. The plate I invariably make of gold with a narrow tongue supporting the pin, extending a short distance along the under side of the plate, the whole being of minimum size and weight. In taking this impression I have never found it necessary to resort to the unique procedure described by Dr. Swain, *i. e.*, of holding the artificial palate in position with a string passed through the nose. A wire bent to enter the pin-hole of the palate and held with one hand, the plaster can, with no great difficulty, be laid on with a spatula by the other and without danger of changing the correct position of the artificial velum. After the arch has been filled and the plaster hard, it can be finished in the ordinary way of taking impressions, using a tray with a flat bottom.

Misfortunes Never Come Singly.—"I don't know what will come next," a good old lady was heard to remark the other day. "John is near-sighted, you know, and his wife hard of hearing, and now they've got a red-headed baby."—*Detroit Free Press.*

## Figures and Tables

**Figure f1:**
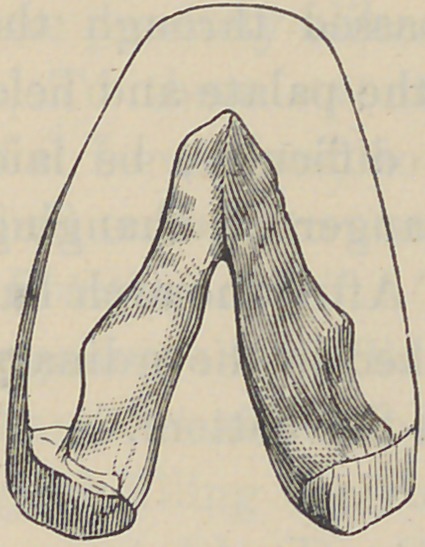


**Figure f2:**